# Structural and Function Correlation of Cone Packing Utilizing Adaptive Optics and Microperimetry

**DOI:** 10.1155/2015/968672

**Published:** 2015-06-08

**Authors:** Dabir Supriya, Mangalesh Shwetha, Kumar Kiran Anupama, Kurian Kummelil Mathew, Tos T. J. M. Berendschot, Jan S. A. G. Schouten, Roopa Bharamshetter, Yadav K. Naresh, Shetty Rohit, Bharath Hegde

**Affiliations:** ^1^Department of Retina, Narayana Nethralaya, No. 121/C, 1st R Block, Rajajinagar, Bangalore 560010, India; ^2^Department of Cataract & Refractive Surgery, Narayana Nethralaya, No. 121/C, 1st R Block, Rajajinagar, Bangalore 560010, India; ^3^Department of Cornea & Refractive Surgery, Narayana Nethralaya, No. 121/C, 1st R Block, Rajajinagar, Bangalore 560010, India; ^4^Department of Ophthalmology, Maastricht University, P.O. Box 616, 6200 MD Maastricht, Netherlands; ^5^Forus Health Pvt. Ltd, No. 2234, 23rd Cross Road, Banashankari Stage II, Banashankari, Bangalore, India

## Abstract

*Aim*. To assess the functional aspects of cone mosaic and correlate cone packing with retinal sensitivity utilizing microperimetry in emmetropes at different eccentricities. *Methods*. Twenty-four healthy volunteers underwent microperimetry (MAIA Centervue, Italy) and assessment of photoreceptors using adaptive optics retinal camera, rtx1 (Imagine Eyes, Orsay, France), at 2 and 3 degrees from the foveal centre in 4 quadrants: superior, inferior, temporal, and nasal. Data was analyzed using SPSS version 17 (IBM). Spearman's correlation tests were used to establish correlation between mean cone packing density and retinal sensitivity at different quadrants. *Results*. Thirteen females and 11 males (age range 20–40 years) were included. The cone density was found to be significantly different among all quadrants (temporal = 25786.68/mm^2^ ± 4367.07/mm^2^, superior = 23009.35/mm^2^ ± 5415.81/mm^2^, nasal = 22838.09/mm^2^ ± 4166.22/mm^2^, and inferior = 21097.53/mm^2^ ± 4235.84/mm^2^). A statistical significance (*P* < 0.008) was found between orthogonal meridians, that is, temporal, nasal (48624.77/mm^2^)> superior, inferior (44106.88/mm^2^). A drop in retinal sensitivity was observed as the eccentricity increased (*P* < 0.05). It was also found that as cone packing density decreased retinal sensitivity also decreased (*P* < 0.05) in all quadrants. This was observed at both 2 and 3 degrees. *Conclusion*. It is of crucial importance to establish normative variations in cone structure-function correlation. This may help in detection of subtle pathology and its early intervention.

## 1. Introduction

Adaptive optics (AO) is emerging as an objective tool in assessment of the architecture of the photoreceptor layer of retina. It can be used to quantify the cone mosaic including the density and packing arrangements. Studying the cone mosaic shows different reflectance patterns with wide temporal and spatial variations. Multiple AO systems have described this variation in the cone reflectivity to be secondary to differences in the phase of phototransduction, length of the outer segment, disc shedding, wavelength of the light, and so forth [1–3].

By just studying the cone mosaic, we are unable to assess the functional aspect of a visible cone and correlate whether a visible cone is a functional cone.

Our study aims to assess the functional aspects of the cone mosaic and correlate the cone packing with the retinal sensitivity utilizing microperimetry (MAIA) in emmetropes at different eccentricities.

## 2. Subjects

Twenty-four healthy volunteers were included in the study after an informed consent was obtained, approved by the institutional review board and in adherence to the tenets of Helsinki declaration. Inclusion criteria were emmetropia or best correct visual acuity of 20/20 or better with astigmatism less than 2 diopters (as assessed by the Tonoref RKT-7000 autorefractometer, Nidek). Subjects with ocular or systemic diseases or previous eye surgery were excluded from the study.

## 3. Methods

All subjects underwent objective refraction, noncontact biometry (IOL master; Carl Zeiss Meditec, Germany) for axial length, and microperimetry (MAIA Centervue-100809). A compact AO retinal camera prototype, the rtx1 (Imagine Eyes, Orsay, France), was used to image the photoreceptor layer. Core components of the apparatus include a Shack-Hartmann wavefront sensor (HASO 32-eye; Imagine Optics, Orsay, France), a deformable mirror (MIRAO 52; Imagine Optics), and a low-noise high-resolution camera (Roper Scientific, Tucson, AZ). AO imaging sessions were conducted after dilating the pupils with 1 drop each of 0.5% tropicamide and 10% phenylephrine hydrochloride. Stable fixation was maintained by having the patient look at the system's inbuilt target moved by the investigator to predetermined coordinates. The patient was instructed to fixate at 0°, 2°, and 3° eccentricity along all the four quadrants, superior, inferior, nasal, and temporal retina. A series of 40 frames, 4° field size, was captured at each of the above retinal locations. After acquisition, a program provided by the manufacturer correlated and averaged the captured image frames to produce a final image [4]. At each site a sampling window square of 100 microns width was chosen avoiding blood vessels. Cone counting software created on MATLAB by Imagine Eyes was used to process the images and calculate the cone density (cones/mm^2^) and spacing. The axial length was entered into the automated software to account for differences in magnification.

**Figure 1:**
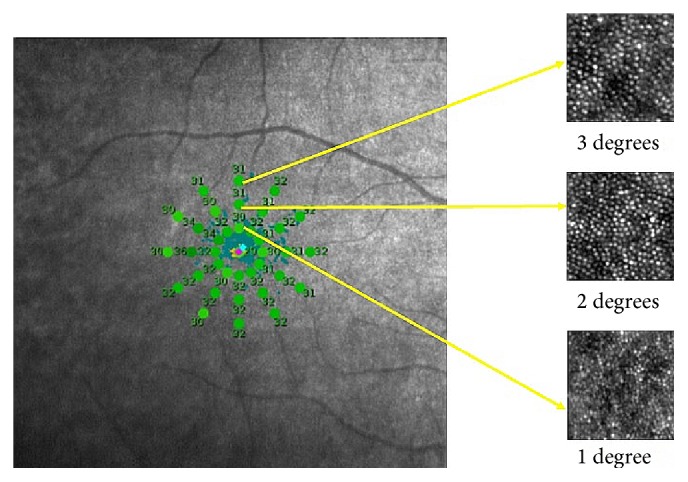
The retinal sensitivity on the MAIA image being correlated to the cone packing density at 1, 2, and 3 degrees from the fovea and 4 quadrants (superior, inferior, temporal, and nasal).

Macular integrity was tested with MAIA, a nonmydriatic, near infrared, line SLO scanning laser ophthalmoscope with high frequency eye tracker, a third generation automated macular perimeter with normative database and a statistical analysis module. An expert or detailed threshold test takes about 4–7 min for each eye and was performed. The grid selected was 37 point stimuli covering the central 6 degrees with 25 *μ*m stimulus size, that is, Goldmann III. The threshold values at radius of 2 degrees and 3 degrees from the fovea were considered in all the 4 quadrants: superior, inferior, nasal, and temporal. The Goldmann size III target subtends 0.431° of visual angle and represents 0.123 mm (0.431°  ∗  0.286 mm/°) on the retina and an area of 0.012 mm^2^ [5]. The sampling window that we have used with the AO image processing is 0.1 mm, and hence the correlation has the potential for fine retinotopic precision as seen in Figure 1.

## 4. Statistical Analysis

The data collected was analyzed using SPSS version 17 (IBM). Spearman's correlation tests were used to establish the correlation between the mean cone packing density and the retinal sensitivity at the different quadrants. To look for possible differences between MAIA threshold values at the different quadrants and eccentricities, a Linear Mixed Models analysis (LMM) was performed with subject ID as grouping factor and cone density, eccentricity, and quadrant and their interaction term as covariates. The LMM procedure expands the general linear model so that the data are permitted to exhibit correlated and nonconstant variability. The LMM analysis, therefore, provides the flexibility of modeling not only the means of the data but their variances and covariances as well. LMM handle data where observations are not independent, as in this study. That is, LMM correctly models correlated errors, whereas procedures in the general linear model family usually do not [6, 7]. *P* values smaller than 0.05 were considered to be significant.

## 5. Results

**Figure 2:**
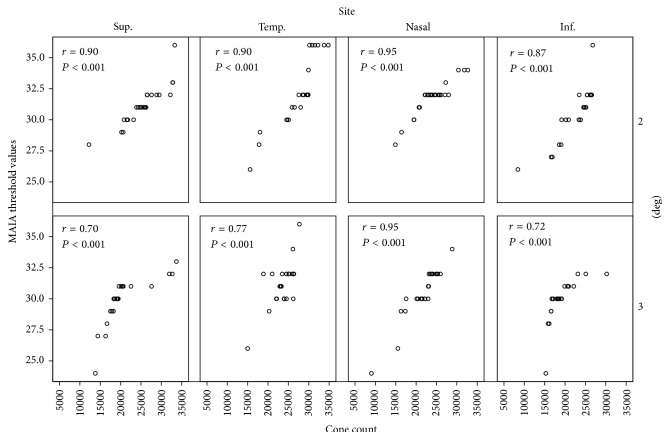
Scatter plot showing correlation between cone density and average threshold at 4 quadrants, both at 2 and 3 degrees.

**Figure 3:**
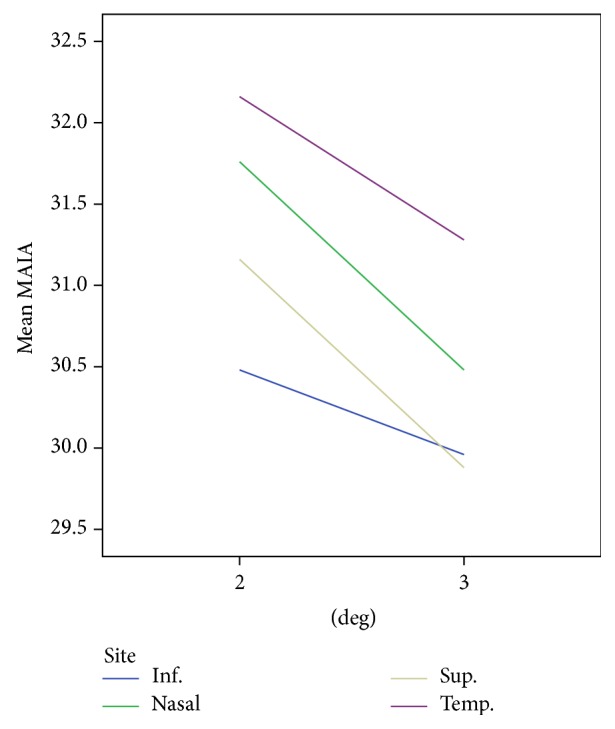
Mean MAIA thresholds at 2 and 3 degrees for the different quadrants.

Twenty-four subjects were included in the study. The study group comprised of 13 females and 11 males between the ages of 20 and 40 years.  Figure 2 shows MAIA threshold values as a function of cone density for the different quadrants. The Pearson correlation coefficient, *r*, was significant for all sites (*P* < 0.001). The cone density was found to be significantly different among all the four quadrants (temporal: 25786 ± 4367 mm^−2^, superior: 23009 ± 5415 mm^−2^, nasal: 22838 ± 4166 mm^−2^, and inferior: 21097 ± 4235 mm^−2^). A statistical significance (*P* < 0.008) was found between the orthogonal meridians, that is, temporal, nasal > superior, inferior (temporal + nasal = 48624 mm^−2^ > superior + inferior = 44106 mm^−2^).  Figure 3 shows mean MAIA thresholds at 2 and 3 degrees for the different quadrants. A drop in the retinal sensitivity was observed as the eccentricity increased. LMM analysis revealed that MAIA threshold values differed significantly between the four quadrants (temporal = 32.2 ± 2.7 dB, superior = 31.2 ± 1.6 dB, nasal: 31.8 ± 1.4 dB, and inferior: 30.5 ± 2.1 dB, *P* = 0.001) and also between the two eccentricities (see Figure 3, *P* = 0.01).

## 6. Discussion

With the advent of adaptive optics leading to compensation of higher order aberrations, the in vivo imaging of the photoreceptor mosaic is now a reality. The challenge now comes in assessing the correlation of the cone mosaic with their functioning. It is interesting to understand whether areas with dense cone packing are associated with higher retinal sensitivities. Establishing the normative database in emmetropes is essential before we understand pathology. This may be useful in establishing the functional correlates of photoreceptor mosaic structure in patients with macular disease who develop central scotomas due to various diseases like age related macular degeneration. They can then be coached to prefer a certain peripheral part of retina to fixate with, depending on the cone density and retinal sensitivity at that area [8, 9]. Even in children after squint surgeries, they may be trained to develop fixation by utilizing the structure-function knowledge of the retinal areas.

There has been a lot of literature on the use of microperimetry alone to find the preferential retinal locus in patients with central macular disease and they have found it to be differing with respect to the task assigned to the patient [8–12]. This however does not happen in normal adults where the preferential retinal locus is fixed. Hence it may be possible to rehabilitate these patients once we understand the areas in which relative structural photoreceptor loss has led to relative functional loss.

The Goldmann size III target has the diameter which subtends 0.431° of visual angle which corresponds to the sampling window of the adaptive optics and hence the correlation has the potential for fine retinotopic precision.

Our study shows that when the mean cone packing density decreased with increasing eccentricity, the corresponding retinal sensitivity also decreased (*P* < 0.05).

The limitations of our study are that we have used a flood illuminated AO camera and not an AO-SLO based microperimetry system [13] which would have better localizing. Also multifocal electrophysiology would have been a more objective tool to analyze the macular function but the costs of tests were a limiting factor.

This study may help establish a sensitive outcome measure to evaluate the safety and efficacy of newer treatment modalities like stem cell therapy and gene therapy in the management of genetic retinal disorders.

## 7. Conclusion

Understanding the correlation between the anatomy of a structure and its function is crucial to plan management of any disease. Knowing the variations in a healthy population helps us analyze pathology better.

## References

[1] Jonnal R. S., Besecker J. R., Derby J. C. (2010). Imaging outer segment renewal in living human cone photoreceptors. *Optics Express*.

[2] Pircher M., Zawadzki R. J., Evans J. W., Werner J. S., Hitzenberger C. K. (2008). Simultaneous imaging of human cone mosaic with adaptive optics enhanced scanning laser ophthalmoscopy and high-speed transversal scanning optical coherence tomography. *Optics Letters*.

[3] Pallikaris A., Williams D. R., Hofer H. (2003). The reflectance of single cones in the living human eye. *Investigative Ophthalmology and Visual Science*.

[4] Lombardo M., Serrao S., Ducoli P., Lombardo G. (2013). Influence of sampling window size and orientation on parafoveal cone packing density. *Biomedical Optics Express*.

[5] Garway-Heath D. F., Caprioli J., Fitzke F. W., Hitchings R. A. (2000). Scaling the hill of vision: the physiological relationship between light sensitivity and ganglion cell numbers. *Investigative Ophthalmology and Visual Science*.

[6] Jansen I., Beunckens C., Molenberghs G., Verbeke G., Mallinckrodt C. (2006). Analyzing incomplete discrete longitudinal clinical trial data. *Statistical Science*.

[7] Laird N. M., Ware J. H. (1982). Random-effects models for longitudinal data. *Biometrics*.

[8] Calabrèse A., Bernard J.-B., Hoffart L. (2011). Wet versus dry age-related macular degeneration in patients with central field loss: different effects on maximum reading speed. *Investigative Ophthalmology and Visual Science*.

[9] Nilsson U. L., Frennesson C., Nilsson S. E. G. (2003). Patients with AMD and a large absolute central scotoma can be trained successfully to use eccentric viewing, as demonstrated in a scanning laser ophthalmoscope. *Vision Research*.

[10] Shima N., Markowitz S. N., Reyes S. V. (2010). Concept of a functional retinal locus in age-related macular degeneration. *Canadian Journal of Ophthalmology*.

[11] Pacella E., Pacella F., Mazzeo F. (2012). Effectiveness of vision rehabilitation treatment through MP-1 microperimeter in patients with visual loss due to macular disease. *Clinica Terapeutica*.

[12] Fujita K., Yuzawa M. (2003). Preferred retinal locus in patients with age-related macular degeneration. *Nippon Ganka Gakkai zasshi*.

[13] Tuten W. S., Tiruveedhula P., Roorda A. (2012). Adaptive optics scanning laser ophthalmoscope-based microperimetry. *Optometry and Vision Science*.

